# Dual-energy computed tomography imaging with megavoltage and kilovoltage X-ray spectra

**DOI:** 10.1117/1.JMI.11.2.023501

**Published:** 2024-03-04

**Authors:** Giavanna Jadick, Geneva Schlafly, Patrick J. La Rivière

**Affiliations:** University of Chicago, Department of Radiology, Chicago, Illinois, United States

**Keywords:** computed tomography, dual-energy, estimation theory, basis material decomposition, megavoltage imaging, simulation

## Abstract

**Purpose:**

Single-energy computed tomography (CT) often suffers from poor contrast yet remains critical for effective radiotherapy treatment. Modern therapy systems are often equipped with both megavoltage (MV) and kilovoltage (kV) X-ray sources and thus already possess hardware for dual-energy (DE) CT. There is unexplored potential for enhanced image contrast using MV-kV DE-CT in radiotherapy contexts.

**Approach:**

A single-line integral toy model was designed for computing basis material signal-to-noise ratio (SNR) using estimation theory. Five dose-matched spectra (3 kV, 2 MV) and three variables were considered: spectral combination, spectral dose allocation, and object material composition. The single-line model was extended to a simulated CT acquisition of an anthropomorphic phantom with and without a metal implant. Basis material sinograms were computed and synthesized into virtual monoenergetic images (VMIs). MV-kV and kV-kV VMIs were compared with single-energy images.

**Results:**

The 80 kV-140 kV pair typically yielded the best SNRs, but for bone thicknesses >8  cm, the detunedMV-80 kV pair surpassed it. Peak MV-kV SNR was achieved with ∼90% dose allocated to the MV spectrum. In CT simulations of the pelvis with a steel implant, MV-kV VMIs yielded a higher contrast-to-noise ratio (CNR) than single-energy CT and kV-kV DE-CT. Without steel, the MV-kV VMIs produced higher contrast but lower CNR than single-energy CT.

**Conclusions:**

This work analyzes MV-kV DE-CT imaging and assesses its potential advantages. The technique may be used for metal artifact correction and generation of VMIs with higher native contrast than single-energy CT. Improved denoising is generally necessary for greater CNR without metal.

## 1 Introduction

When imaging for radiation therapy, high soft-tissue contrast is essential. Tumors must be accurately imaged at all stages: before treatment for dose calculations, during treatment for patient positioning, and after treatment for cancer monitoring.^1^ The current imaging standard in radiation therapy is single-energy (SE) computed tomography (CT). While SE-CT provides clear contrast between bone and soft tissue, it has difficulty distinguishing variations within tissue structures. To achieve a sufficient contrast-to-noise ratio (CNR) to identify a tumor on a soft-tissue background, a higher patient dose may be necessary. With the advent of modern image-guided radiation therapy (IGRT) and its daily imaging requirements, individual image doses are in danger of accumulating over the course of treatment up to the scale of a therapy dose fraction.^2^ Thus, there is interest in finding methods that achieve sufficient image quality for IGRT with reduced dose.

One potential solution could be on-board dual-energy (DE) CT. In diagnostic imaging, DE-CT is routinely applied to enhance contrast. By acquiring multiple spectral data points, sufficient information is available for the calculation of basis material images and virtual monoenergetic images (VMIs). With carefully chosen contrast agents and X-ray spectra, image quality may be greatly improved using DE-CT.^3^^–^^6^

To achieve multiple simultaneous CT acquisitions, DE-CT imaging requires special hardware such as multiple source-detector arrays, energy-discriminating detectors, or fast kV-switching sources. A system lacking this hardware may also perform DE-CT by means of repeat acquisitions, but this method can suffer from misalignment or motion artifacts. Modern radiation therapy treatment systems are often already equipped with dual X-ray sources: a megavoltage (MV) source for treatment and a kilovoltage (kV) source for on-board imaging. Given this readily available equipment, there exists an unexplored potential for enhancing image quality in the context of radiation therapy using MV-kV DE-CT.^6^^,^^7^

A variety of methods are being explored to improve image contrast in radiation therapy settings without delivering excess patient dose. CT is currently the only accepted imaging modality for three-dimensional treatment planning dose calculations, as it provides empirical information on electron density and atomic composition. Unfortunately, X-ray imaging modalities like CT require increased dose to yield sufficient soft-tissue contrast for this purpose and additionally suffer from a lack of functional information and, in some cases, metal artifacts. To overcome these drawbacks, there has been recent interest in magnetic resonance (MR) and positron emission tomography (PET) imaging at different stages of the treatment planning process.^1^^,^^8^ MR- and PET-linac systems are currently being developed and even introduced in some clinics.^9^^,^^10^ Though these systems show promise, they are emerging and expensive, and CT imaging still remains necessary for dose calculation. Combined MV-kV imaging has the unique advantage of utilizing existing hardware, resulting in a much lower barrier to implementation and the potential to be realized in a shorter time frame. Moreover, MV images have the potential to be acquired during patient treatment, providing dual energy information to augment kV images without necessitating additional dose.^7^^,^^11^^,^^12^

While some prior work has explored ways to combine MV and kV information for various applications, work specifically exploring MV-kV DE-CT is limited. This may be due to the drawbacks of MV images, which can potentially contaminate kV images and reduce image quality if the two are combined naively. Since MeV photons are generally more penetrating than keV photons and have greater dose deposition per photon, MV images typically have lower contrast and greater noise relative to kV images with the same dose.^12^^,^^13^ There are limited situations in which MV imaging alone is sufficient. For example, MV localizers or CT images have been shown to be sufficient for radiotherapy setup verification, which is useful for linacs lacking a kV X-ray source.^14^ The superior quality of kV imaging is still necessary for initial treatment planning.

When strategically implemented, MV information can be combined with kV information to achieve better soft-tissue contrast than could be achieved with either source alone and an equivalent total dose. Various techniques have been described previously.^6^^,^^12^^,^^13^^,^^15^^–^^21^ For example, the greater penetrability of MV photons becomes an advantage when imaging highly attenuating objects; thus, MV data may be synthesized with kV images for metal artifact correction.^18^^,^^21^ Similar methods have been implemented for target tracking during radiotherapy.^16^^,^^17^

Previous work has shown promise for MV-kV DE-CT.^6^^,^^12^^,^^13^^,^^15^^,^^19^^,^^20^ With only partial angular information, combined MV-kV CT images can achieve superior image quality relative to kV or MV alone by inheriting the advantages of each single-energy image: higher contrast from kV images and reduction in streak artifacts from MV images. These techniques utilize 90 deg to 110 deg of data from each spectrum, possibly with a small amount (10 deg to 15 deg) of overlap, then implement either a linear gray scale conversion or histogram mapping of pixels to reconstruct a single kV or MV image with complete angular information. Though these methods improve efficiency by reducing rotation time, the imperfect spectral mapping can cause artifacts and reduce image quality. To our knowledge, previous work focusing specifically on MV-kV DE-CT has not considered datasets with complete angular information in both MV and kV domains.

The precise conditions for MV-kV DE-CT to yield improved image quality with equivalent dose have not been fully characterized. The experimental nature of this past work, utilizing image quality phantoms, limits the number of data points feasible for robust analysis and optimization of MV-kV DE-CT. These methods typically acknowledge the drawback of greater dose deposition by MeV photons but do not consider the effect of dose distribution between MV and kV spectra. They observe that image quality is best when MV beams penetrate more highly attenuating material inserts, but they image only a small number of material inserts within image quality phantoms. A more complete analysis should assess image quality for a continuum of material attenuations and spectral dose distributions.

In this work, we implement the analytical method proposed by Roessl and Herrmann,^22^ which uses estimation theory to characterize basis material signal-to-noise ratio (SNR) along a single-line integral with DE X-rays. This method has not yet been applied to MV-kV imaging. With this technique, we are able to survey a wide range of parameters without experimental measurements, facilitating more robust characterization of situations in which MV-kV imaging provides superior image quality and quantification of the degree of improvement. This similarly allows us to assess whether it is possible to achieve equivalent image quality and reduced dose with MV-kV imaging. We consider three variables: spectral combination, dose allocation between the two spectra, and material composition. To gauge utility of MV-kV DE-CT in a more clinically realistic setup, we also extend this model to a CT raytracing simulation with parameters informed by the single-line optimization.

## 2 Methods

Two models were developed for quantifying basis material image quality: a toy model using a single-line integral with a two-material object and a fan-beam CT simulation with a computational anthropomorphic phantom. The single-line model was used to maximize basis material SNR as a function of spectral dose allocation for an object with various bone thicknesses. These results were used to inform the simulated CT imaging task.

### 2.1 Signal Detection Framework

The detected signal λ with each spectrum i was calculated as

$$\lambda_{i}=\int_{E_{\min}}^{E_{\max}}D(E)\mathrm{Poi}\{I_{i}(E)T(E)\eta (E)dE\}$$ (1)

where E is the energy, $I_{i}(E)$ is the incident X-ray spectrum (photons per energy), T(E) is the object transmission function, η(E) is the detective efficiency function, D(E) is the detector response function, and the notation $\mathrm{Poi}\{\overset{¯}{x}\}$ indicates generation of a realization of a Poisson random variable with mean $\overset{¯}{x}$. In Eq. (1), the argument of the Poisson noise is the mean number of photons of energy E stopped by the detector. This indicates that the signal noise model is compound Poissonian weighted by D(E).

Transmission T(E) was computed as the line integral attenuation through the object of interest

$$T(E)=e^{-\int_{L}d\ell \mu (x,y;E)},$$ (2)

where ℓ is the distance along the given ray L and μ(x,y;E) is the linear attenuation coefficient of the material at location (x,y) evaluated at energy E.

**Fig. 1:**
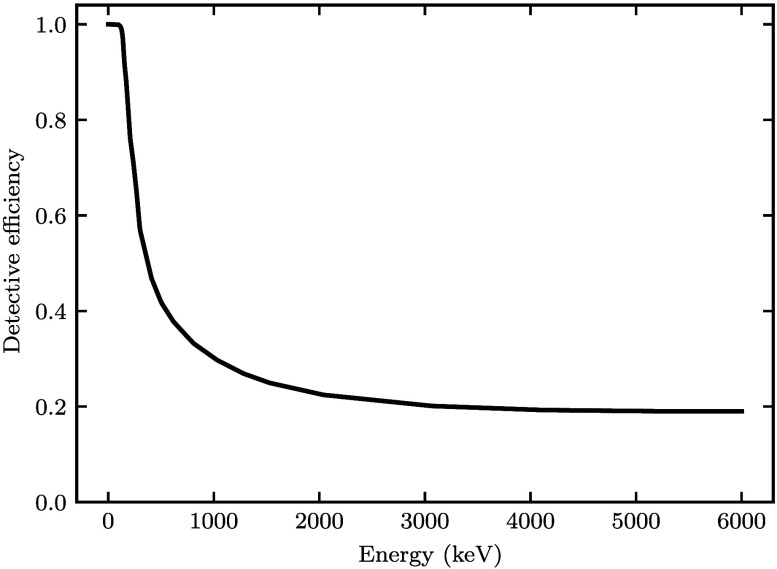
The modeled detective efficiency function η(E).

An energy-integrating detector (EID) was modeled [D(E)=E] with detective efficiency η(E) as shown in Fig. 1. The detective efficiency function was computed to yield performance consistent with that of a previously described high-detective quantum efficiency (DQE) xenon gas detector.^23^^,^^24^ Such detectors have been implemented on commercial tomotherapy units (Accuray Inc., Sunnyvale, CA) for fan-beam MV CT imaging.^25^^,^^26^

#### 2.1.1 Input spectra

**Fig. 2:**
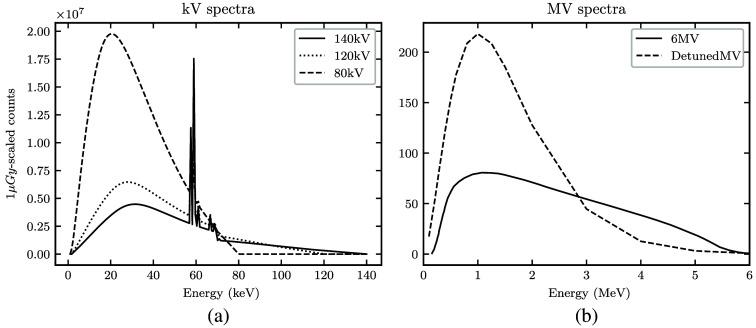
(a) and (b) The five spectra with magnitude scaled to deliver 1  μGy dose.

Five polychromatic spectra $I_{i}(E)$ were modeled (Fig. 2). Three kV spectra were chosen to represent common diagnostic CT options (80, 120, and 140 kV). The two MV spectra were modeled after a typical treatment beam (6 MV) and a treatment beam with energy detuned to below 3 MV for imaging (detunedMV).^27^^,^^28^ To ensure a valid basis for comparison, the flux of each spectrum was scaled to deliver the same dose to the center of a 40-cm diameter water cylinder (depth $d_{w}=20\;\mathrm{cm}$) under the condition of charged particle equilibrium

$$\text{Dose}(d_{w})=\int_{E_{\min}}^{E_{\max}}dEI_{i}(E)e^{-\mu_{w}(E)d_{w}}{\left(\frac{\mu_{\mathrm{en}}(E)}{\rho }\right)}_{w}E,$$ (3)

where $\mu_{w}(E)$ is the linear attenuation coefficient of water and $[\mu_{\mathrm{en}}(E)/\rho {]}_{w}$ is the mass energy absorption coefficient of water.^29^

### 2.2 Model 1: Single-Line Integral Through A Two-Material Object

Basis material SNR was computed using an estimation theory framework for a single ray incident on a two-material object of ICRU tissue and bone with densities $\rho_{\text{tissue}}=1.00\;g/{\mathrm{cm}}^{3}$ and $\rho_{\text{bone}}=1.85\;g/{\mathrm{cm}}^{3}$.^22^^,^^30^ The tissue thickness was fixed at $t_{\text{tissue}}=40\;\mathrm{cm}$ and the bone thickness was varied from $t_{\text{bone}}=1$ to 10 cm. All MV-kV and kV-kV spectral pairs were considered, yielding nine DE combinations. The total single-line dose allocated to both spectra was set to 1  μGy. For a typical CT acquisition with near 1000 projection views, this would sum to a dose of 1 mGy. SNR was characterized as a function of dose allocation r in 1% increments (from 1% to 99%), where the high-energy spectrum was rescaled by r and the low-energy spectrum by 1−r.

The SNR for each basis material j was defined as the ratio of the true mass thickness ($A_{j}\equiv \rho_{j}t_{j}$) to the square root of the Cramér–Rao lower bound (CRLB) on variance

$${\mathrm{SNR}}_{j}=\frac{A_{j}}{\sigma_{A_{j}}}.$$ (4)

The CRLB was found from the Fisher information F using the known relation $\sigma_{A_{j}}^{2}\geq F_{jj}^{-1}$.^31^ The signal noise model, which was energy-weighted compound Poisson, was approximated as a Gaussian with mean and variance matching the first two moments of the true distribution.^4^ The mean of each measurement is simply $\lambda_{i}$ as in Eq. (1), and the corresponding variance $\sigma_{i}^{2}$ is

$$\sigma_{i}^{2}=\int_{E_{\min}}^{E_{\max}}dED^{2}(E)I_{i}(E)T(E)\eta (E).$$ (5)

Thus, each DE acquisition (i=1,2) yields a Fisher information matrix with elements in terms of $\lambda_{i}$ and $\sigma_{i}^{2}$ and their partial derivatives with respect to the true mass thicknesses A

$$F_{jk}=\underset{i=1,2}{\sum }\frac{1}{\sigma_{i}^{2}}\frac{\partial \lambda_{i}}{\partial A_{j}}\frac{\partial \lambda_{i}}{\partial A_{k}}+\frac{1}{2}\underset{i=1,2}{\sum }\frac{1}{(\sigma_{i}^{2}{)}^{2}}\frac{\partial \sigma_{i}^{2}}{\partial A_{j}}\frac{\partial \sigma_{i}^{2}}{\partial A_{k}},$$ (6)

where i is the spectral index and j,k are the basis material indices.^22^

### 2.3 Model 2: Fan-Beam CT of an Anthropomorphic Phantom

To assess whether MV-kV DE-CT may provide advantages in clinical imaging scenarios, the signal detection framework was extended from the single-line model to a fan-beam CT geometry with 1200 views, 800 detector channels, and a fan angle of 47 deg. A single 360 deg rotation was simulated for each acquisition. Beam transmission through a computational anthropomorphic phantom, the extended cardiac torso (XCAT), was calculated.^32^ The phantom had dimensions of 512×512 with $1\;{\mathrm{mm}}^{2}$ pixels. Path lengths through each pixel were determined using Siddon’s algorithm for the exact radiological path through a CT array.^33^

**Fig. 3:**
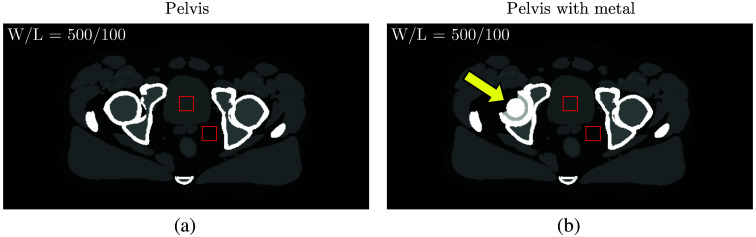
The computational phantom imaged (a) without and (b) with a metal hip replacement as indicated by the arrow. Contrast levels correspond to noiseless 80 keV VMIs. In the modeled hip prosthesis, the femoral head and outer shell were either titanium or stainless steel, and the inner lining was PMMA. CNR was computed using the delineated ROIs, and RMSE was evaluated within the phantom.

**Fig. 4:**
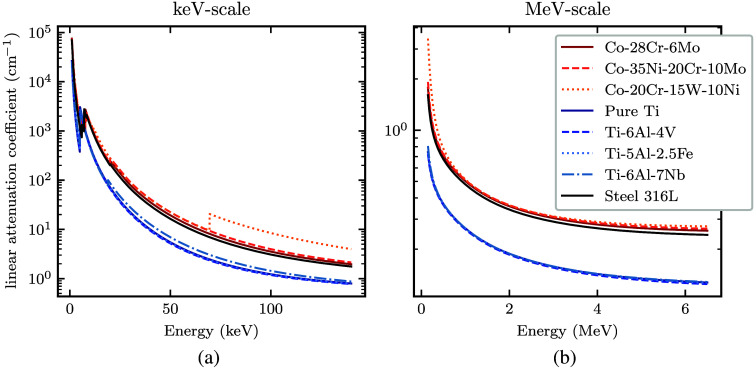
Linear attenuation coefficients of metals common in modern hip prostheses (cobalt-chromium alloys, titanium alloys, and surgical-grade stainless steel) in (a) the keV energy range and (b) the MeV energy range.

Since MeV photons are generally more penetrating than keV photons, the effect of high attenuation was considered by imaging the pelvis region with and without a metal hip replacement (Fig. 3).^34^ A hip prosthesis comprises three primary components: a femoral head, a cap or lining, and an outer shell. Each part must be wear-resistant, biocompatible, and capable of bearing high static and dynamic loads; tradeoffs among these factors inform the proper choice of materials for each patient’s unique presentation.^35^ Metals have traditionally been and continue to be the central component of implants for total hip arthroplasty.^34^^–^^36^ Contemporary metals of choice are stainless steel, titanium alloys, or cobalt-chromium (Co-Cr) alloys.^35^ Figure 4 shows the linear attenuation coefficients of these materials, indicating that titanium alloys are generally less attenuating than Co-Cr alloys and stainless steel. To represent these typical attenuation categories, we modeled two types of metal implants: (i) commercially pure titanium and (ii) surgical grade stainless steel for both the femoral head and shell with a PMMA lining.

The total dose of each DE-CT acquisition was set to 10 mGy. Sinograms were generated for two spectral pairs, “MV-kV” (9 mGy detunedMV and 1 mGy 80 kV) and “kV-kV” (5 mGy 140 kV and 5 mGy 80 kV). Each sinogram pair was decomposed into ICRU tissue and bone basis materials using a Gauss–Newton algorithm.^37^ While basis material decomposition may occur in either the sinogram or image domain, the sinogram-domain decomposition was chosen because of its advantage of ameliorating beam hardening artifacts. The basis material sinograms were then reconstructed into basis material images (BMIs) using fan-beam filtered back projection (FFBP) including a general sinc window with cutoff frequency at 80% of the Nyquist frequency.^38^ These BMIs correspond to the densities of the materials $\rho_{i}$. The reconstructed images had a matrix size of 512×512 and field-of-view of 50 cm. VMIs were generated at various energies $E_{0}$ as a linear combination of the BMIs

$$\mathrm{VMI}(E_{0})=\rho_{1}{\left[\frac{\mu (E_{0})}{\rho }\right]}_{1}+\rho_{2}{\left[\frac{\mu (E_{0})}{\rho }\right]}_{2},$$ (7)

where $[\mu (E_{0})/\rho {]}_{j}$ is the known mass attenuation coefficient of basis material j at the energy $E_{0}$.

For comparison, three 10 mGy single-energy (SE) acquisitions were also generated (80, 120, and 140 kV) and reconstructed using the same FFBP algorithm. As the DE VMIs have the advantage of ameliorating beam-hardening effects, a two-material beam-hardening correction (BHC) was applied to the SE images. A water correction was first applied in the sinogram domain using a fourth-degree polynomial remapping based on known attenuation characteristics.^38^ A bone correction was then applied in the image domain by generating an artifact-only image from a thresholded “bone” image, which was linearly combined with the original image to create the final corrected image.^38^ This allowed us to compare dose-matched and artifact-corrected SE- and DE-CT, which is more fair than comparison of each 10 mGy DE-CT acquisition with its noisier constituent <10  mGy SE-CT simulations. Better image quality with equivalent dose also indicates that dose can be reduced while maintaining equivalent image quality.

To evaluate image quality, CNR was computed in each 10 mGy SE-CT image and DE-CT VMI using measurements from the ROIs delineated in Fig. 3. CNR was defined as

$$\mathrm{CNR}=\frac{\left|{\mathrm{Avg}[\mathrm{ROI}}_{1}]-{\mathrm{Avg}[\mathrm{ROI}}_{2}]\right|}{\sqrt{{\mathrm{Var}[\mathrm{ROI}}_{1}]+{\mathrm{Var}[\mathrm{ROI}}_{2}]}},$$ (8)

where ${\mathrm{ROI}}_{1}$ is the signal and ${\mathrm{ROI}}_{2}$ is the background. We also examined contrast and noise individually, defined as the numerator and denominator of Eq. (8), respectively.

To evaluate the accuracy, the VMIs were registered to the input phantom, and root-mean-square-error (RMSE) was computed relative to the monoenergetic ground truth,

$$\mathrm{RMSE}=\sqrt{\mathrm{Avg}[(\mathrm{VMI}-\mathrm{XCAT}{)}^{2}]}.$$ (9)

Note that SE-CT measurements are not energy-dependent, whereas CNR, RMSE, VMI, and XCAT include implicit energy dependence from each monoenergetic evaluation.

## 3 Results

### 3.1 Model 1: Single-Line Integral

**Fig. 5:**
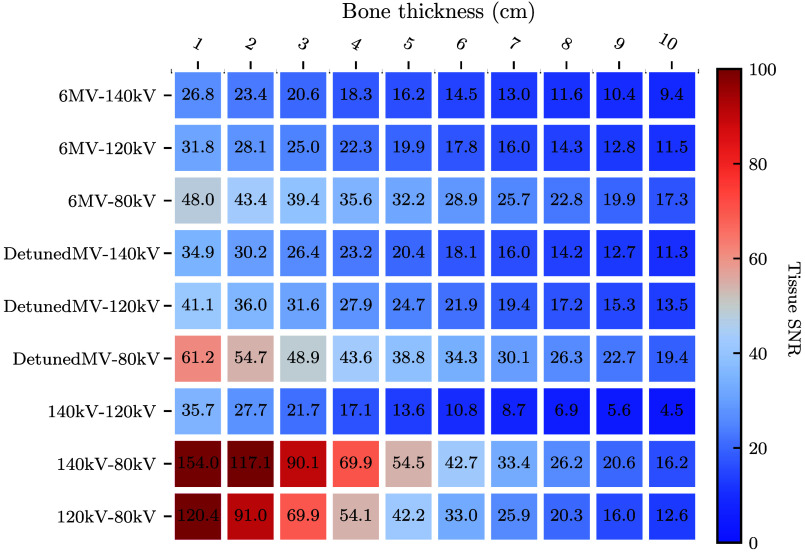
Heatmap of peak tissue SNR as a function of bone thickness for all dual-energy spectral combinations.

**Fig. 6:**
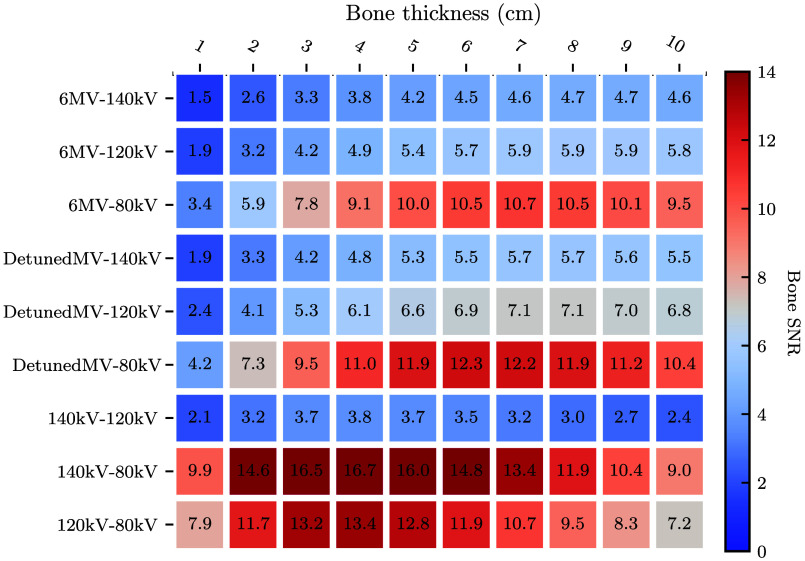
Heatmap of peak bone SNR as a function of bone thickness for all dual-energy spectral combinations.

To identify the most promising spectral pairs, Figs. 5 and 6 present heatmaps of peak tissue and bone SNR, respectively, for each spectral combination and the ten bone thicknesses. Peak SNR was found from the curve of SNR as a function of dose allocation r. For both basis materials, the 140 kV-80 kV pair yields the highest SNRs overall. The detunedMV-80 kV pair yields the highest SNRs of the MV-kV pairs. These two spectral pairs will be the focus of further analysis (“kV-kV” and “MV-kV,” respectively). Tissue SNR is maximized for both pairs with 1 cm bone, and bone SNR is maximized for kV-kV at 4 cm bone and MV-kV at 6 cm bone.

**Fig. 7:**
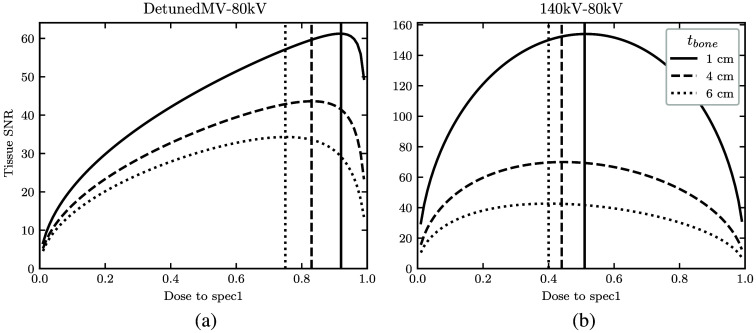
With 1, 4, or 6 cm bone thickness, curve of bone SNR as a function of dose allocated to the high-energy spectrum for (a) the detunedMV-80 kV and (b) 140 kV-80 kV spectral pairs.

Looking at optimal spectral dose distribution, Fig. 7 shows tissue basis material SNR as a function of high-energy dose allocation r for three different bone thicknesses. Based on the peaks in the two heatmaps, bone thicknesses of 1, 4, and 6 cm were chosen. Table 1 lists the coordinates of the peak basis material SNR for all spectral combinations with 1 cm bone. The MV-kV curve is skewed toward allocating a greater proportion of dose to the MV spectrum, peaking at r=0.92, 0.83, and 0.75 for 1, 4, and 6 cm, respectively. The kV-kV curve favors a more equal dose distribution, peaking at r=0.51, 0.44, 0.40. As bone thickness increases, SNR is maximized by increasing the dose allocated to the low-energy spectrum.

**Table 1:** Peak coordinates ($r_{\max},{\mathrm{SNR}}_{\max}$) as a function of dose allocation for both basis materials and all spectral pairs with a bone thickness of 1 cm.

Spectral pair	Tissue		Bone	
	$r_{\max}$	SNR $\left(r_{\max}\right)$	$r_{\max}$	SNR $\left(r_{\max}\right)$
6MV-80kV	0.93	47.99	0.89	3.36
6MV-120kV	0.93	31.79	0.91	1.86
6MV-140kV	0.93	26.79	0.91	1.49
detunedMV-80kV	0.92	61.23	0.87	4.24
detunedMV-120kV	0.92	41.11	0.89	2.39
detunedMV-140kV	0.91	34.88	0.89	1.93
140kV-80kV	0.51	154.02	0.45	9.89
140kV-120kV	0.51	35.72	0.50	2.09
120kV-80kV	0.50	120.38	0.45	7.94

**Fig. 8:**
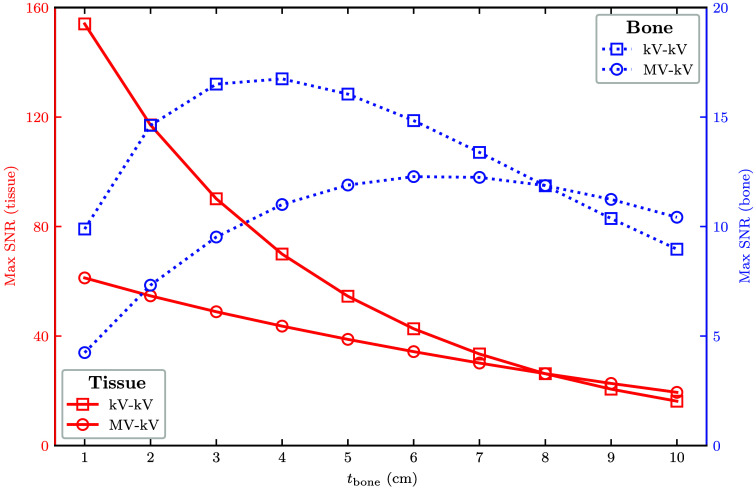
Maximum SNR for tissue (solid lines, left scale) and bone (dashed lines, right scale) for the 140 kV-80 kV (kV-kV, square marker) and detunedMV-80 kV (MV-kV, circle marker) spectral combinations as a function of bone thickness.

To assess the effect of increasing object attenuation, Fig. 8 shows peak SNR as a function of bone thickness for the MV-kV and kV-kV pairs. At low bone thicknesses, kV-kV SNR is higher than MV-kV SNR for both basis materials. The tissue SNR monotonically decreases with bone thickness, and the bone SNR reaches a maximum at a thickness of 4 cm (kV-kV) or 6 cm (MV-kV). As bone thickness increases, the difference between the two curves decreases, and at 8 cm bone, the MV-kV curves intersect the kV-kV curves and begin to yield higher SNRs. This is due to the more rapid SNR drop-off of kV-kV imaging at high bone thickness.

### 3.2 Model 2: Fan-Beam CT

**Fig. 9:**
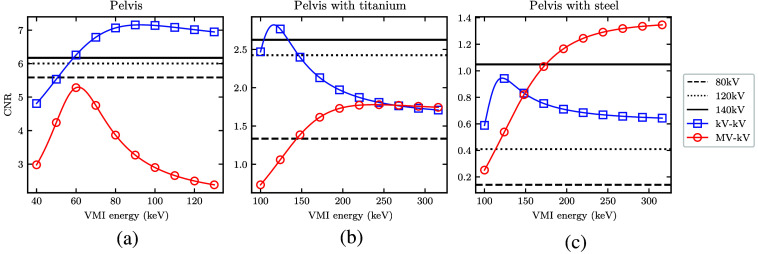
(a)–(c) CNR in the detunedMV-80kV (MV-kV) and 140kV-80kV (kV-kV) VMIs as a function of energy, with horizontal lines at the CNR of each kV single-energy CT acquisition. The total dose of the dual-energy scans was equivalent to the dose of the single-energy scans (10 mGy).

To quantify image quality in the simulated CT images, Fig. 9 shows CNR in the VMIs of the pelvis phantom with and without the metal hip replacement (titanium or steel). CNR was measured as a function of VMI energy, and horizontal lines were drawn at the fixed CNR for each dose-matched, beam-hardening corrected kV SE-CT image. These represent thresholds above which DE-CT may yield improved image quality for a given imaging task. The DE-CT peak CNRs and SE-CT CNR thresholds with and without BHC are listed in Table 2. In the pelvis and pelvis with titanium, single-energy CNR improves considerably with BHC. With steel, the single-energy images are too noisy for the BHC algorithm implemented to sufficiently reduce streaking artifacts. With BHC, single-energy CT is able to outperform DE-CT in certain cases. For the pelvis and pelvis with titanium, kV-kV DE-CT yields the best CNR, and the peak MV-kV CNR is lower than the SE-CT threshold. For the more highly attenuating steel, MV-kV DE-CT yields the best CNR, and the peak kV-kV CNR is below the SE-CT threshold. Without BHC, the SE-CT threshold is surpassed by the peak CNR of both MV-kV and kV-kV acquisitions for all three phantoms.

**Table 2:** For each phantom, maximum CNR measured in the MV-kV and kV-kV VMIs, and single-energy CNRs with and without a BHC. For each dual-energy acquisition, the VMI energy corresponding to the maximum CNR is indicated in parentheses. For each single-energy spectrum, the change in CNR with BHC is indicated in parentheses.

Acquisition	Pelvis CNR	Pelvis with titanium CNR	Pelvis with steel CNR
MV-kV	5.30 (61 keV)	1.78 (239 keV)	1.35 (315 keV)
kV-kV	7.16 (93 keV)	2.82 (116 keV)	0.94 (122 keV)
80 kV	4.16	0.97	0.02
80 kV (BHC)	5.59 (+1.43)	1.33 (+0.36)	0.14 (+0.12)
120 kV	4.79	1.47	0.46
120 kV (BHC)	6.00 (+1.21)	2.42 (+0.95)	0.41 (–0.05)
140 kV	4.94	1.63	0.73
140 kV (BHC)	6.17 (+1.23)	2.63 (+1.00)	1.05 (+0.32)

**Fig. 10:**
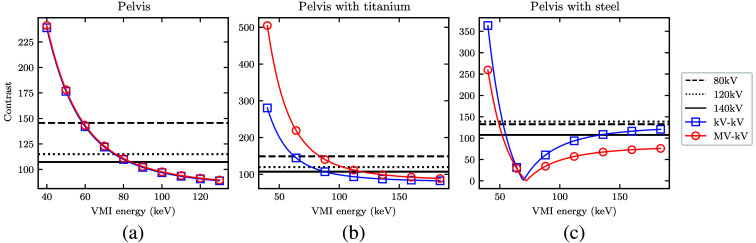
(a)–(c) Measured contrast (numerator of the CNR) in the detunedMV-80 kV (MV-kV) and 140 kV-80 kV (kV-kV) VMIs as a function of energy, with horizontal lines at the value of each dose-matched kV single-energy CT acquisition.

**Fig. 11:**
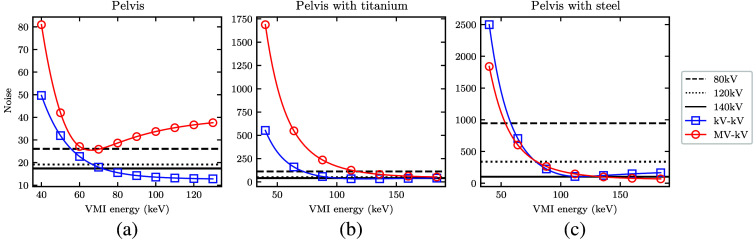
(a)–(c) Measured noise (denominator of the CNR) in the detunedMV-80 kV (MV-kV) and 140 kV-80 kV (kV-kV) VMIs as a function of energy, with horizontal lines at the value of each dose-matched kV single-energy CT acquisition.

To better understand the CNR results, Figs. 10 and 11 show the separate contrast and noise values within the overall ratio. At low monoenergies, both MV-kV and kV-kV VMIs yield the highest native contrast, as expected. This is accompanied by a high noise level, especially in the MV-kV VMIs. This is likely due to the relative up-weighting of the 80 kV acquisition with only 10% dose allocation at low VMI monoenergies. At high monoenergies, DE-CT VMIs generally have less noise and lower contrast than the dose-matched SE-CT images. The relative rate of change of the two factors differs for each phantom, as indicated in the conglomerate ratios shown in Fig. 9.

**Fig. 12:**
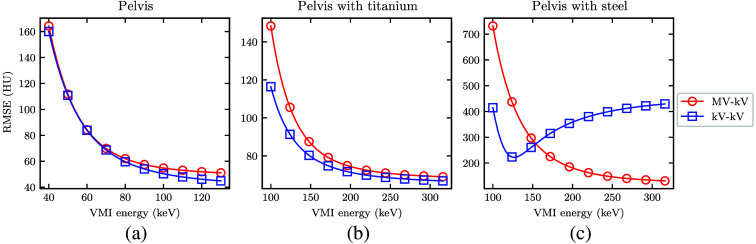
(a)–(c) RMSE in the detunedMV-80kV (MV-kV) and 140kV-80kV (kV-kV) VMIs as a function of energy. RMSE was calculated relative to the monoenergetic ground truth XCAT.

To quantify accuracy of the simulated CT images relative to the known ground truth, Fig. 12 shows RMSE for each DE-CT VMI. Generally, RMSE appears to decrease with greater VMI energy. Depending on the imaging setup, this lower error may come at the cost of higher CNR (Fig. 9). For the pelvis without metal, the minimum RMSE is 44.9 HU for kV-kV and 51.1 HU for MV-kV DE-CT. The smallest error overall is achieved with kV-kV DE-CT, though the difference relative to MV-kV is small (+6.2 HU). With metal, the RMSE measurements are much larger, likely due to the overall higher noise and possible streaking artifacts. For the titanium case, the scale of the error is similar, with a minimum RMSE of 66.9 HU for kV-kV and 69.0 HU for MV-kV DE-CT (+2.1 HU). For the steel case, MV-kV yields the lowest error; the minimum RMSE is 222.2 HU for kV-kV and 130.8 HU for MV-kV DE-CT (−91.4  HU). The discrepancy in accuracy is also reflected in the contrast measurements shown in Fig. 10. Ideally, MV-kV and kV-kV acquisitions would yield VMIs with identical contrast values that match the known ground truth for each monoenergy. The contrast curves match well for the pelvis alone but diverge with a titanium or steel implant.

Appendix shows the simulated CT images. Each DE-CT acquisition includes the constituent single-energy images, BMIs, and example VMIs. The dose-matched, beam-hardening corrected SE-CT images are also shown.

## 4 Discussion

This work presents an evaluation of MV-kV DE-CT imaging, which could be realized using existing hardware in radiotherapy settings. We approached this task beginning with an estimation theory framework for calculating SNR along a single ray and then expanding to a simulated fan-beam CT acquisition of an anthropomorphic phantom. By comparing MV-kV DE-CT images with dose-matched single-energy kV CT images and diagnostic kV-kV DE-CT images, we were able to assess whether MV-kV imaging could be used to achieve improved image quality or reduced dose relative to current clinical standards. Even small dose reductions could potentially compound over many IGRT fractions to result in a significantly lower total dose from imaging over a patient’s course of treatment.

Our single-line integral estimation theory method requires at least two input X-ray spectra to compute a theoretical upper bound on the achievable basis material SNR.^22^ We use this method to compare MV-kV DE-CT to diagnostic kV-kV DE-CT. However, in a realistic radiation therapy scenario, the available imaging option would likely be a single-energy scan (either MV or kV) rather than kV-kV DE-CT since existing treatment systems lack dual kV X-ray sources. The availability of dual MV and kV X-ray sources is a unique practical advantage of MV-kV DE-CT. While the kV-kV single-line integral SNR calculations provide useful context for the MV-kV SNRs, our CT simulation allows us to make a more clinically relevant comparison of single-energy kV CT with DE MV-kV DE-CT. We also simulated kV-kV DE-CT images for context with the single-line integral SNRs.

Of the nine spectral pairs considered (six MV-kV and three kV-kV), the best basis material SNRs were found using the 140 kV-80 kV spectral pair. This is expected, as this spectral pair is commonly used for diagnostic DE-CT imaging since it maximizes the energy separation between high- and low-energy spectra given the peak voltages conventionally available with modern X-ray tubes.^3^ Of the MV-kV DE pairs, the best SNRs were found using the detunedMV-80 kV pair and the second-best with the 6MV-80 kV pair. As the detuned beam has a lower effective energy than the treatment beam, it is expected to yield better image quality due to its higher detective efficiency and native contrast. However, the 6 MV treatment beam has the potential to be used for acquiring images simultaneously with radiation therapy treatment, whereas the detuned beam could only be used for imaging before or after treatment.^11^ In this work, we chose to focus on the detuned beam. This choice provides a metric of the best achievable MV-kV CT image quality using beams currently available on therapy treatment systems, which is useful for an assessment of clinical viability.

One unique consideration of this study was the effect of dose allocation between the two spectra in a DE acquisition. We found that spectral dose distribution has a considerable effect on MV-kV image quality. As kV X-ray spectra have relatively similar effective energy and detective efficiency, dose allocation may not be a typical consideration for diagnostic DE-CT. Single-ray basis material SNR peaked with ∼90% dose allocated to the detunedMV spectrum or 50% dose to the 140 kV spectrum when paired with the 80 kV low-energy spectrum. The exact optimal dose allocation varied depending on the basis material and bone thickness. The asymmetric SNR-versus-dose allocation curve is a unique aspect of MV-kV DE-CT, likely owing to the greater dose deposition per photon with energy in the MeV range. In general, the optimal r was slightly higher for tissue SNR than for bone SNR. For both spectral pairs, r also decreased with increasing bone thickness. This relation was steeper for the MV-kV DE-CT pair. These results suggest spectral dose allocation is an important consideration for clinical implementation of MV-kV CT. Though past work assessing combined MV-kV image quality often draws comparisons with single-energy acquisitions, there is lack of a consideration of dose variations.^12^^,^^15^^,^^19^^–^^21^ A fair comparison of MV-kV DE-CT with single-energy CT should utilize dose-matched acquisitions with optimal spectral dose distributions depending on object composition. Our method provides one such way of carrying out this optimization.

For the fan-beam CT simulations, we chose the dose distribution between spectra using the single-ray approximations, with a 90:10 MV-kV distribution and 50:50 kV-kV distribution in each ray. Optimal dose allocation is more complex for CT than it is for a single ray. A CT acquisition comprises thousands of line integrals over many views and detector channels, each passing through a distinct section of the anatomy with varying attenuation. For rays including greater bone thickness, our results indicate that more doses should be allocated to the low-energy spectrum. We expect that image quality in our DE-CT simulations could be improved with more thorough dose allocation optimization. Traditional CT imaging uses bowtie filtration to reduce the dose allocated to more peripheral detector angles, producing more similar spectrum magnitudes after attenuation and, consequently, more uniform noise in each channel.^39^ Tube current modulation (TCM) may additionally be implemented to modulate the dose delivered at each view angle, equalizing the noise in each projection.^40^ The single-line model could be used to inform a DE-CT TCM algorithm that incorporates a method for optimizing dose allocation to each spectrum at each view. Since the optimal dose allocation as a function of bone thickness varied more quickly for the detunedMV-80kV pair than it did for the diagnostic 140 kV-80 kV pair, MV-kV DE-CT image quality may especially benefit from such an algorithm, and it is worth considering for future applications.

In the single-line integral model, tissue SNR monotonically decreased as a function of bone thickness. Bone SNR peaked at 4 cm for the 140 kV-80 kV pair and 6 cm for the detunedMV-80 kV pair. At low bone thicknesses, the SNR achieved with the kV-kV pair was higher than that with the MV-kV pair. This is more relevant for most imaging scenarios, especially at antero-posterior or posto-anterior CT view angles. However, at 8 cm bone thickness and greater, the MV-kV pair yields the highest SNRs. With the greater attenuation due to high bone thickness, the higher penetrability of the MeV photons becomes more beneficial as kV images begin to suffer from photon starvation. This effect has been utilized in other work for artifact correction around highly attenuating objects, namely metal implants.^18^^,^^21^ Our findings corroborate this effect. For this reason, we explored DE-CT BMI quality with and without metal implants in the XCAT phantom.^32^

The simulated CT images show an advantage of MV-kV DE-CT over dose-matched single-energy kV CT and kV-kV DE-CT when imaging the pelvis with a steel hip prosthesis. This suggests potential utility of the technique for metal artifact correction. When imaging the pelvis alone or with the less attenuating titanium implant, kV-kV DE-CT yielded the best image quality. Though both DE acquisitions could be used to generate low-energy VMIs with greater native contrast, the higher noise in the MV-kV acquisitions translated into a lower CNR than the dose-matched, artifact-corrected single-energy CT images of the pelvis without metal. With either titanium or steel hip prosthesis, as expected, image quality suffered for all SE-CT acquisitions. The severity of metal artifacts was especially apparent in the constituent kV images with the more highly attenuating steel implant, and MV-kV VMIs yielded the best CNR and RMSE. For the relatively less attenuating titanium implant, the kV-kV and MV-kV CNRs converge above 200 keV. However, visual inspection of the 300 keV VMIs (Appendix) indicates that the images have very different textures; the kV-kV image [Fig. 15(f)] has better contrast but a residual streak artifact, whereas the MV-kV image [Fig. 16(f)] is noisier but with more uniform texture. Likewise, the dose-matched, artifact-corrected SE-CT images produce a higher CNR than the MV-kV VMIs in the pelvis alone or with titanium, but there are some residual beam-hardening artifacts that affect the noise texture (Fig. 19). Our metric of image quality, CNR, is unable to capture this difference in texture, and it is not clear which image would be universally preferable. Further work might explore whether such texture differences are clinically significant.

DE acquisitions can bypass the need for a separate BHC by using a material decomposition algorithm in the sinogram domain. We found that BHC is necessary for single-energy CNRs to exceed the peak MV-kV CNRs. We did not apply any post-processing to the DE-CT VMIs. Looking at the MV-kV pelvis-only images (Fig. 14), it appears that the main contributor to VMI noise is the MV acquisition. Even with 90% of the total dose allocated to the MV spectrum, it has a lower incident flux than the kV spectrum due to the higher dose deposition per photon in the MeV energy range. DE-CT has the unique potential to mitigate the high noise in the MV acquisitions with a multi-channel denoising technique that utilizes the shared edge structure of images acquired with different spectra.^41^^,^^42^ Though not implemented in our work, such a method could be particularly useful for MV-kV DE-CT, as kV images have much lower noise and sharper edges to inform denoising in the MV images. Commercial imaging equipment may implement a proprietary denoising algorithm, as well. Given the potential for MV-kV imaging to generate low-energy VMIs with high native contrast, such methods might be explored to test whether MV noise could be sufficiently reduced to produce a higher CNR than that of single-energy CT for more general imaging scenarios. Nevertheless, these simulation results demonstrate the value of MV information for metal artifact correction and VMI generation.

Other work has similarly drawn comparisons between combined MV-kV images and single-energy kV images.^12^^,^^15^ Li et al.^15^ observed that MV-kV VMIs of an image quality phantom can yield improved CNR relative to single-energy kV images with proper selection of virtual monoenergy. They found low monoenergy is preferable for low-density material inserts, and conversely, high monoenergy is preferable for high-density inserts. This trend matches our findings of VMI CNR as a function of monoenergy in the pelvis with and without metal. Similarly, Yin et al.^12^ measured comparable or better contrast in aggregate MV-kV reconstructions compared to kV alone, depending on the material. It is relevant to note that these studies utilized a partial-angle acquisition technique and did not account for dose distribution, which is distinct from our method.

We chose to model a fan-beam, high-DQE, EID with an efficiency near 20% in the MeV energy range (Fig. 1). This emulates the xenon gas detectors that have been used for MV CT on commercial tomotherapy units (Accuray Inc., Sunnyvale, CA).^23^^–^^26^ Prior research indicates that a DQE(0) of at least 20% is needed for sufficient image quality without delivering excessive dose to the patient, with non-negligible dose concerns emerging due to daily imaging of patients for modern IGRT.^2^^,^^43^ However, conventional linear accelerators typically acquire MV images using a cone-beam CT geometry and flat-panel electronic portal imaging device (EPID) with a much lower detective efficiency of 1% to 2%.^44^ Thus, it is of interest to consider the applicability of our work in the context of conventional cone-beam CT. MV imaging technology is rapidly evolving, and new EPIDs are being introduced with higher detective efficiency, improved spatial resolution, and reduced quantum noise. A variety of promising techniques are being explored, including multi-layered scintillators, novel scintillating materials, and direct conversion detectors.^43^^–^^47^ New EPIDs have been developed with measured DQE(0) of 6.7%,^44^ 8.0%,^45^ 9.7%,^46^ and 22%.^43^ One application of that research demonstrated that single-energy MV CT images acquired with a four-layer EPID have superior image quality relative to MV and kV images acquired with a single-layer detector in the presence of titanium and aluminum implants.^48^ Our results support these findings in the context of DE MV-kV CT for steel artifact correction. Furthermore, as noted, MV-kV DE-CT has the unique potential to compensate for low MV counting efficiency with a multi-channel denoising technique. Thus, we expect our work is also relevant for cone-beam MV-kV DE-CT with either an emerging high-DQE EPID or multi-channel denoising.

Diagnostic kV imaging technology is also advancing. Photon-counting detectors (PCDs) have recently debuted in clinical CT scanners, offering advantages such as higher spatial resolution, lower quantum noise, and dose reduction with image quality comparable to conventional EIDs.^49^^,^^50^ Spectral PCDs mounted on radiotherapy treatment systems could offer a new avenue for single-shot MV-kV DE-CT by using signal pulse height thresholds to “bin” detected photons into sinograms with different energy spectra using a single X-ray beam. Another ongoing area of research is data-driven DE-CT imaging techniques. Deep learning models have been introduced for the generation of DE images from single-energy data and material decomposition.^51^^,^^52^ This work could potentially be utilized in MV-kV imaging, for example with improved material decomposition algorithms that are trained to account for the large contrast and noise differences.

This work was a simplified theoretical analysis of MV-kV DE-CT, and many limitations could be more realistically modeled in future work. Our compound Poisson noise model neglected X-ray scatter, patient motion, and electronic noise. A real CT acquisition will be affected by these complexities, and a DE-CT system using simultaneous acquisitions will also experience cross-scatter from the two beams. Though the simultaneous acquisition method introduces this additional scatter, it has the advantage of reducing motion artifacts relative to a sequential acquisition method. A Monte Carlo simulation could be additionally implemented to approximate both single-source scatter and dual-source cross-scatter, in order to better weigh the costs and benefits of each technique.^53^^,^^54^ Other work has presented new methods for scatter reduction between MV and kV sources, which could also be considered.^55^ Though we did not account for electronic noise in our detector model, new photon-counting detectors are able to threshold out this noise. This could also be an avenue for future work. For image reconstruction, we implemented a standard filtered back-projection algorithm.^38^ More modern iterative and deep learning methods could be implemented, which may include more advanced noise reduction. Such algorithms likely especially benefit MV CT since MV images tend to be noisier than kV images when dose-matched.

One potential extension of this work would be a more realistic differentiation of the two source-detector arrays. While traditional CT is acquired with a full field-of-view, some work has considered MV images that are truncated by multi-leaf collimators (MLCs).^11^ Future work could simulate or acquire MLC leakage images, especially with a simulated dose plan that opens the MLCs around the tumor. These images would use a higher incident flux, and attenuation through the MLCs would be calculated. Such images may suffer from a limited field-of-view and ring artifacts, for which corrections should be explored. On a linac, imaging systems likely utilize cone-beam CT geometry, which can further suffer from greater scatter and smaller field-of-view relative to fan-beam CT.^56^ However, it is important to assess these practical constraints in relation to the standard single-energy CT images. Different detector materials and geometries should be considered for the MV and kV systems. In this work, we assumed the same detector was used for all acquisitions, with a fan shape and a fixed number of channels. This matched detector acquisition allows for material decomposition in the sinogram domain, which has the advantage of alleviating beam hardening artifacts. In current clinical settings, the kV source would likely have a larger fan angle and more detector channels. These different geometries will produce sinograms with different angular extents and other properties, likely resulting in different reconstructed image qualities and necessitating implementation of an image-domain material decomposition algorithm. This may affect the resulting image quality. Even so, the results of this initial investigation provide sufficient motivation for future studies with more faithful geometric modeling.

## 5 Conclusion

This work presents an analysis of MV-kV DE-CT imaging. We estimate that basis material SNR is maximized with 90% dose allocated to the MV spectrum. For bone thicknesses greater than 8 cm, SNR is maximized using MV-kV DE-CT. In a simulated CT scan of the pelvis with a stainless steel hip prosthesis, MV-kV VMIs can produce higher CNR and lower RMSE than diagnostic kV-kV VMIs, indicating the potential utility of this technique for metal artifact correction. With pelvis alone or a less attenuating titanium prosthesis, MV-kV DE-CT can generate low-energy VMIs with higher native contrast, but dose-matched single-energy CT yields a better CNR due to lower noise. For MV-kV to outperform single-energy CT in these cases, multi-channel denoising methods, multi-view dose allocation optimization, and improved MV detectors might be explored. These results demonstrate the potential utility of MV-kV DE-CT, more robustly quantify the parameters for optimal implementation, and motivate future experimental investigation into clinical applications.

## 6 Appendix: Simulated CT Images

**Fig. 13:**
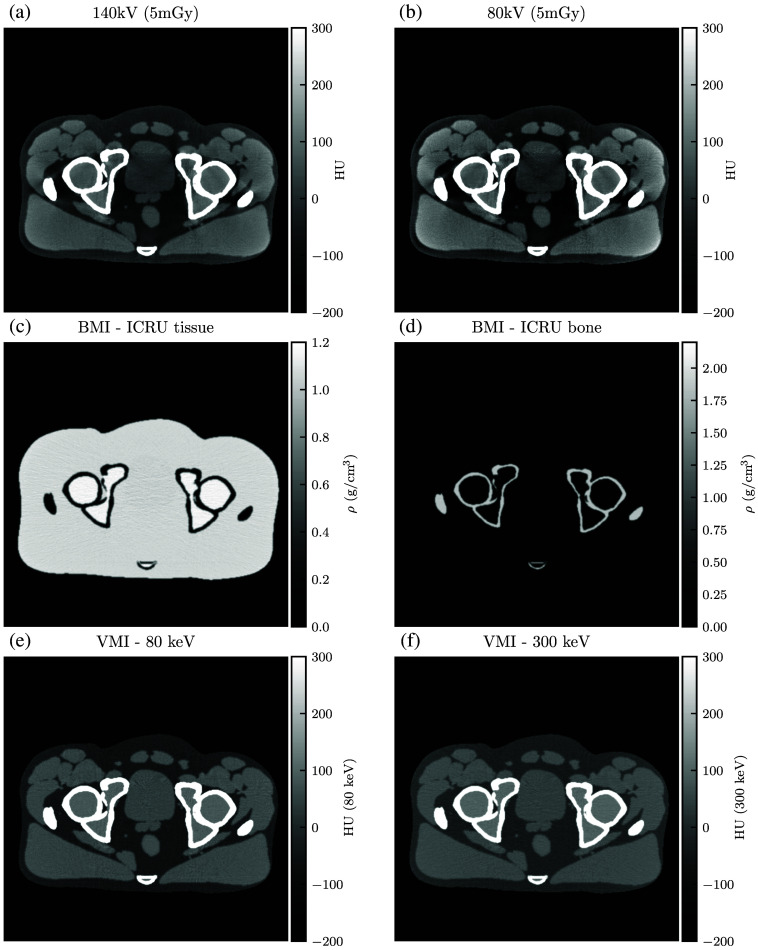
(a) and (b) Single-energy CT images, (c) and (d) BMIs, and (e) and (f) VMIs for the 140 kV-80 kV DE-CT acquisition of the pelvis with no hip replacement. The VMIs correct the beam hardening visible in the single-energy CT images.

**Fig. 14:**
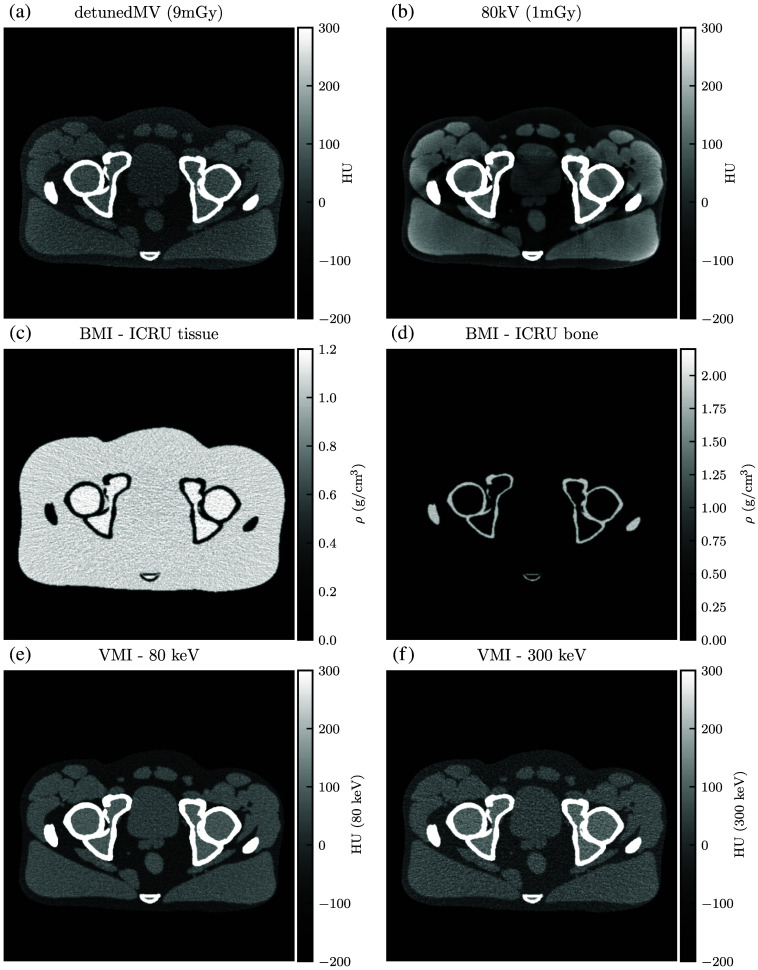
(a) and (b) Single-energy images, (c) and (d) BMIs, and (e) and (f) VMIs for the detunedMV-80 kV DE-CT acquisition of the pelvis with no hip replacement. VMI synthesis corrects for the beam hardening in the 80 kV single-energy image but results in increased noise from the detunedMV single-energy image.

**Fig. 15:**
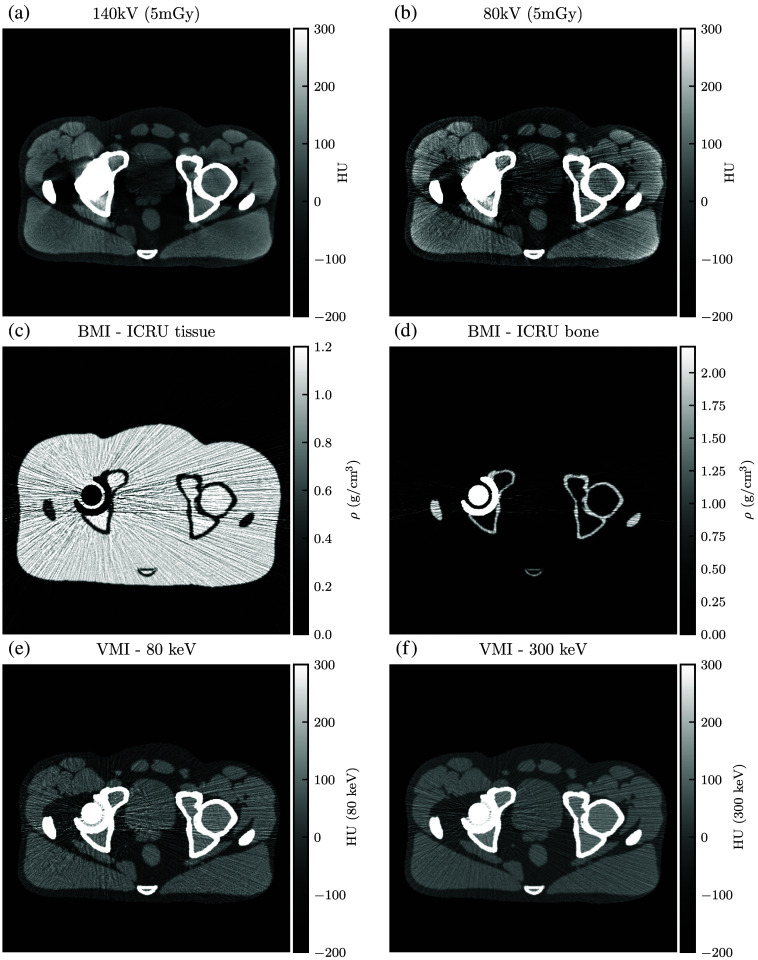
(a) and (b) Single-energy images, (c) and (d) BMIs, and (e) and (f) VMIs for the 140 kV-80 kV DE-CT acquisition with titanium hip replacement. In the VMIs, the beam hardening artifact of the single-energy images has been corrected, but there is residual streaking.

**Fig. 16:**
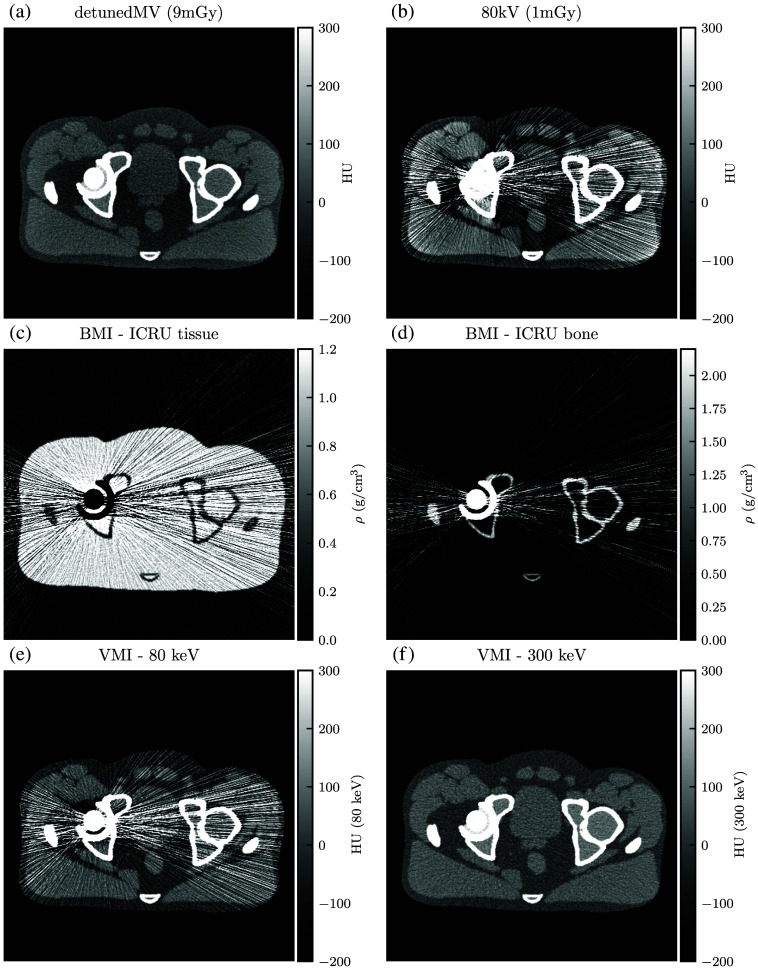
(a) and (b) Single-energy images, (c) and (d) BMIs, and (e) and (f) VMIs for the detunedMV-80 kV DE-CT acquisition with titanium hip replacement. The 300 keV VMI does not display streaking or beam hardening artifacts, but it shows a similar noise profile as the detunedMV single-energy image.

**Fig. 17:**
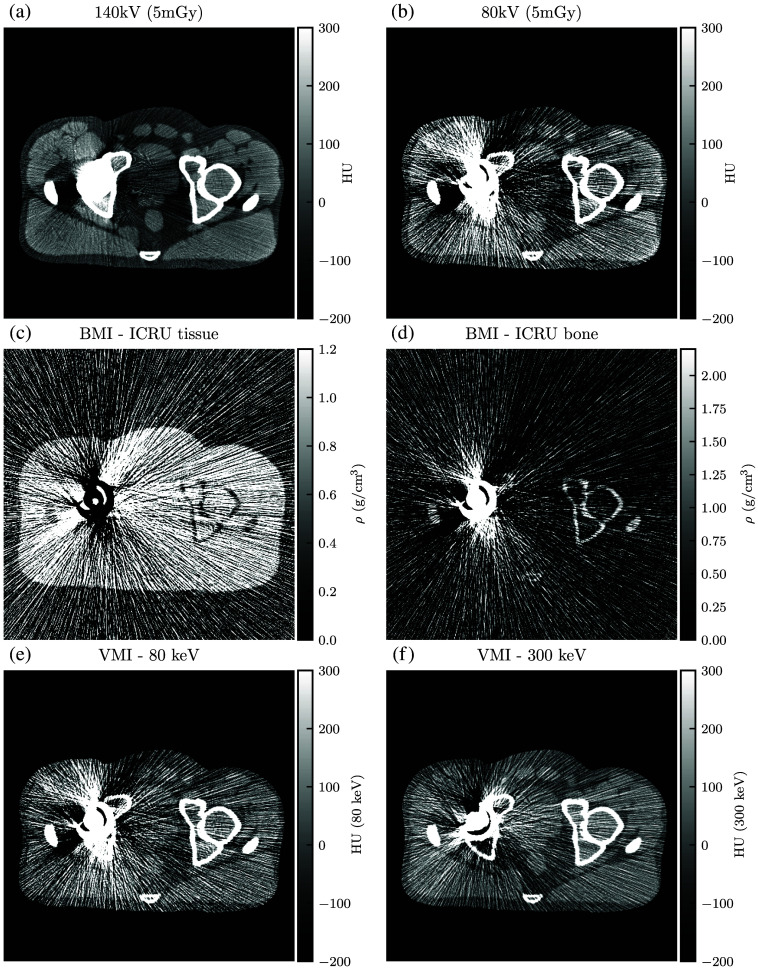
(a) and (b) Single-energy images, (c) and (d) BMIs, and (e) and (f) VMIs for the 140 kV-80 kV DE-CT acquisition with stainless steel hip replacement. Both single-energy images and VMIs suffer from severe streaking artifacts.

**Fig. 18:**
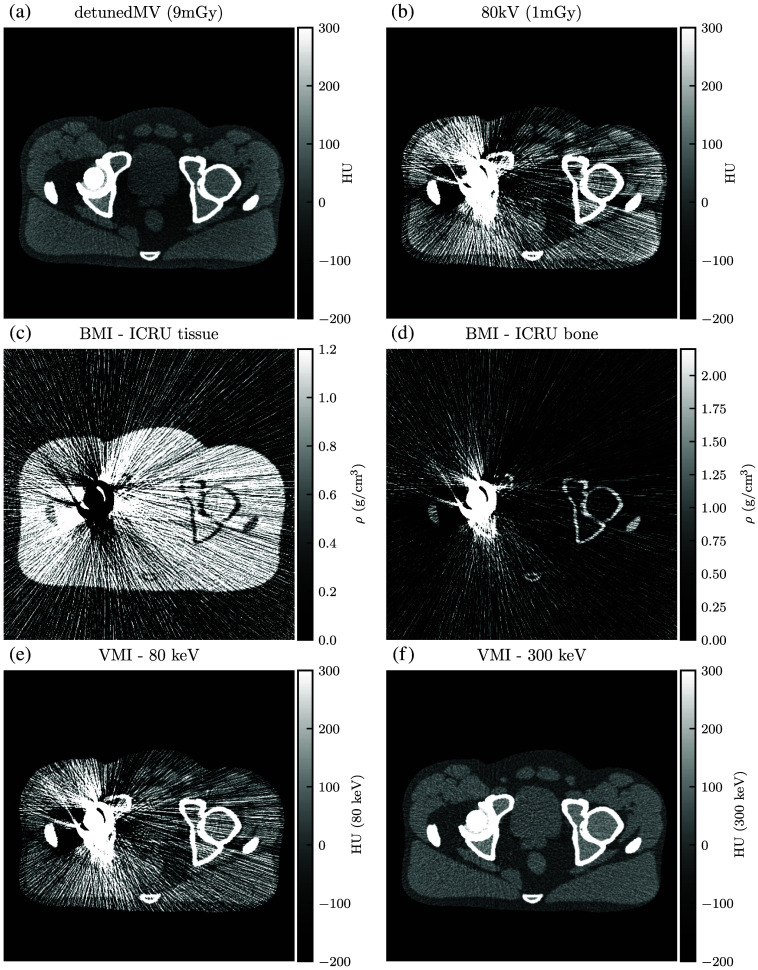
(a) and (b) Single-energy images, (c) and (d) BMIs, and (e) and (f) VMIs for the detunedMV-80 kV DE-CT acquisition with stainless steel hip replacement. The 300 keV VMI highly alleviates the streaking artifact.

**Fig. 19:**
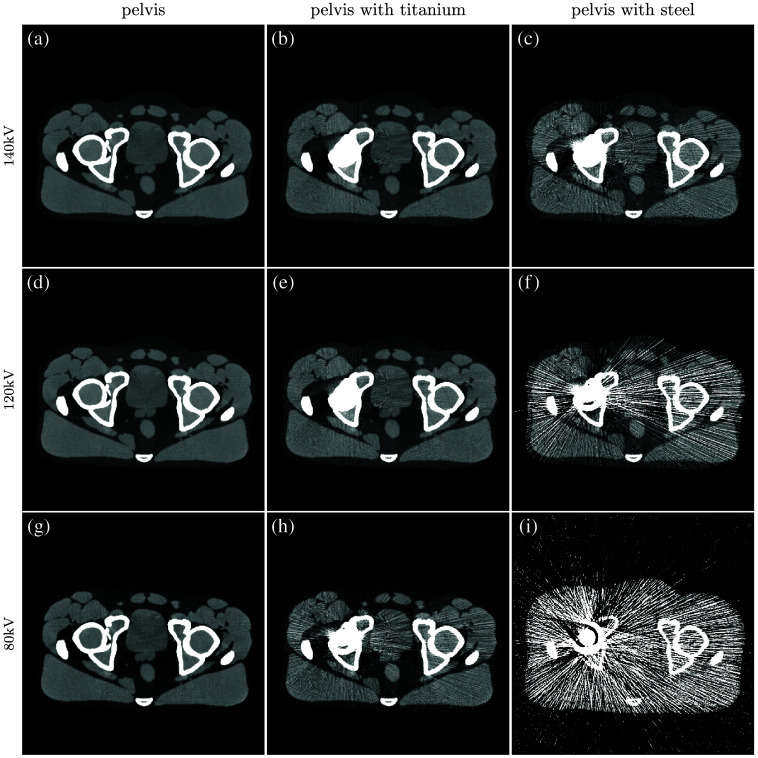
(a)–(i) The dose-matched, beam-hardening corrected SE-CT images for each phantom (column) and kV spectrum (row). Images are shown with a window width of 500 HU and level of 50 HU.

For qualitative analysis, Figs. 13–18 show a sampling of simulated SE-CT images, BMIs, and VMIs used for our analysis, providing complementary visual information for the measurements in Figs. 9 and 12. Figures 13 and 14 show the kV-kV and MV-kV DE-CT acquisitions of the pelvis without metal; Figs. 15 and 16 show the images with titanium hip replacement; and Figs. 17 and 18 show the images with stainless steel hip replacement. Figure 19 shows the dose-matched, beam-hardening corrected SE-CT images used for comparison.

Qualitatively, in each constituent kV SE-CT image, there is noticeable beam hardening in the pelvis and severe streaking artifacts with either titanium or stainless steel hip replacement. These effects are more severe for the more highly attenuating metal, stainless steel. The detunedMV SE-CT images display less beam hardening, and although the metal hip replacements increase noise, they do not cause as severe streaking.

In the dose-matched, artifact-corrected SE-CT images used for comparison with DE-CT VMIs (Fig. 19), there is a considerable reduction in noise and beam hardening. In images of the pelvis alone, the artifact is almost entirely corrected. With a titanium implant, there is noticeable improvement but some residual streaking that affects noise texture. With steel, although the cupping artifact is mostly corrected, the streaking artifacts are too severe to be eliminated.

Similarly, with either metal, after basis material decomposition, the kV-kV and MV-kV BMIs both show considerable streaking. In the MV-kV case, this is likely due to artifact contamination from the 80 kV SE-CT image. This contamination improves after synthesis into VMIs. Without metal, both the MV-kV and kV-kV VMIs have visibly good contrast and no beam hardening. With titanium, the 300 keV VMIs for both MV-kV and kV-kV acquisitions improve upon the SE-CT images; kV-kV has less noise but a moderate streaking artifact, whereas MV-kV is slightly noisier but devoid of streaking. With the more attenuating stainless steel, the 300 keV MV-kV VMI has greatly reduced metal artifact contamination compared to the kV-kV VMIs.

## Data Availability

The code used to generate the data in this manuscript can be freely accessed through GitHub at https://github.com/gjadick/dex-single-ray for the single-line integral toy model and https://github.com/gjadick/dex-ct-sim for the fan-beam CT simulation and basis material decomposition.

## References

[1] Pereira G. C. Traughber M. Muzic R. F., “The role of imaging in radiation therapy planning: past, present, and future,” Biomed. Res. Int. 2014, 231090 (2014). 10.1155/2014/231090 24812609 PMC4000658

[2] Alaei P. Spezi E., “Imaging dose from cone beam computed tomography in radiation therapy,” Physics Med. 31, 647–658 (2015). PHYME2 1120-1797 10.1016/j.ejmp.2015.06.003 26148865

[3] Marin D. et al., “State of the art: dual-energy CT of the abdomen,” Radiology 271, 327–342 (2014). RADLAX 0033-8419 10.1148/radiol.14131480 24761954

[r4] Rigie D. S. La Rivière P. J., “Optimizing spectral CT parameters for material classification tasks,” Phys. Med. Biol. 61(12), 4599–4622 (2016). PHMBA7 0031-9155 10.1088/0031-9155/61/12/4599 27227430 PMC5444336

[r5] Kruis M. F., “Improving radiation physics, tumor visualisation, and treatment quantification in radiotherapy with spectral or dual-energy CT,” J. Appl. Clin. Med. Phys. 23(1), e13468 (2022) 10.1002/acm2.13468 34743405 PMC8803285

[6] Sajja S. et al., “Technical principles of dual-energy cone beam computed tomography and clinical applications for radiation therapy,” Adv. Radiat. Oncol. 5, 1–16 (2020). 10.1016/j.adro.2019.07.013 32051885 PMC7004939

[7] Jadick G. La Rivière P., “Optimization of MV-kV dual-energy CT imaging for tomographic therapy,” Proc. SPIE 12463, 124632L (2023). PSISDG 0277-786X 10.1117/12.2653674

[8] van Elmpt W. et al., “Dual energy CT in radiotherapy: current applications and future outlook,” Radiother. Oncol. 119, 137–144 (2016). RAONDT 0167-8140 10.1016/j.radonc.2016.02.026 26975241

[9] Oderinde O. M. et al., “The technical design and concept of a PET/CT linac for biology-guided radiotherapy,” Clin. Transl. Radiat. Oncol. 29, 106–112 (2021). 10.1016/j.ctro.2021.04.003 34258399 PMC8260396

[10] Winkel D. et al., “Adaptive radiotherapy: the Elekta Unity MR-linac concept,” Clin. Transl. Radiat. Oncol. 18, 54–59 (2019). 10.1016/j.ctro.2019.04.001 31341976 PMC6630157

[11] Ruchala K. J. et al., “Megavoltage CT imaging as a by-product of multileaf collimator leakage,” Phys. Med. Biol. 45, N61 (2000). PHMBA7 0031-9155 10.1088/0031-9155/45/7/401 10943938

[12] Yin F.-F. Guan H. Lu W., “A technique for on-board CT reconstruction using both kilovoltage and megavoltage beam projections for 3D treatment verification,” Med. Phys. 32(9), 2819–2826 (2005). MPHYA6 0094-2405 10.1118/1.1997307 16266096

[13] Zhang J. Yin F.-F., “Minimizing image noise in on-board CT reconstruction using both kilovoltage and megavoltage beam projections,” Med. Phys. 34(9), 3665–3673 (2007). MPHYA6 0094-2405 10.1118/1.2768862 17926970

[14] Mackie T. R. et al., “Tomotherapy,” Semin. Radiat. Oncol. 9(1), 108–117 (1999). SRONEO 10.1016/S1053-4296(99)80058-7 10196402

[15] Li H. Liu B. Yin F.-F., “Generation of virtual monochromatic CBCT from dual kV/MV beam projections,” Med. Phys. 40(12), 121910 (2013). MPHYA6 0094-2405 10.1118/1.4824324 24320521

[r16] Luo W. et al., “Analysis of image quality for real-time target tracking using simultaneous kV-MV imaging,” Med. Phys. 35(12), 5501–5509 (2008). MPHYA6 0094-2405 10.1118/1.3002313 19175109

[r17] Mao W. et al., “A fiducial detection algorithm for real-time image guided IMRT based on simultaneous MV and kV imaging,” Med. Phys. 35(8), 3554–3564 (2008). MPHYA6 0094-2405 10.1118/1.2953563 18777916 PMC2809708

[r18] Pearson E. Pan X. Pelizzari C., “Dual-energy (MV/kV) CT with probabilistic attenuation mapping for IGRT applications,” Proc. SPIE 9412, 94125M (2015). PSISDG 0277-786X 10.1117/12.2082068

[r19] Wertz H. et al., “Fast kilovoltage/megavoltage (kVMV) breathhold cone-beam CT for image-guided radiotherapy of lung cancer,” Phys. Med. Biol. 55(15), 4203 (2010). 10.1088/0031-9155/55/15/001 20616405

[r20] Blessing M. et al., “Breath-hold target localization with simultaneous kilovoltage/megavoltage cone-beam computed tomography and fast reconstruction,” Int. J. Radiat. Oncol. Biol. Phys. 78(4), 1219–1226 (2010). IOBPD3 0360-3016 10.1016/j.ijrobp.2010.01.030 20554124

[21] Li H. et al., “Implementation of dual-energy technique for virtual monochromatic and linearly mixed CBCTs,” Med. Phys. 39(10), 6056–6064 (2012). MPHYA6 0094-2405 10.1118/1.4752212 23039644

[22] Roessl E. Herrmann C., “Cramér–Rao lower bound of basis image noise in multiple-energy x-ray imaging,” Phys. Med. Biol. 54(5), 1307 (2009). 10.1088/0031-9155/54/5/014 19190361

[23] Keller H. et al., “Monte Carlo study of a highly efficient gas ionization detector for megavoltage imaging and image-guided radiotherapy,” Med. Phys. 29(2), 165–175 (2002). MPHYA6 0094-2405 10.1118/1.1445414 11865988

[r24] Ruchala K. J. et al., “Megavoltage CT on a tomotherapy system,” Phys. Med. Biol. 44(10), 2597–2621 (1999). PHMBA7 0031-9155 10.1088/0031-9155/44/10/316 10533931

[r25] Meeks S. L. et al., “Performance characterization of megavoltage computed tomography imaging on a helical tomotherapy unit,” Med. Phys. 32, 2673–2681 (2005). MPHYA6 0094-2405 10.1118/1.1990289 16193798

[26] Schombourg K. Bochud F. Moeckli R., “Stability of the Helical TomoTherapy Hi-Art II detector for treatment beam irradiations,” J. Appl. Clin. Med. Phys. 15(6), 119–127 (2014). 10.1120/jacmp.v15i6.4897 PMC5711117 25493514

[27] Jeraj R. et al., “Radiation characteristics of helical tomotherapy,” Med. Phys. 31(2), 396–404 (2004). MPHYA6 0094-2405 10.1118/1.1639148 15000626

[28] Mackie T. R., “History of tomotherapy,” Phys. Med. Biol. 51(13), R427 (2006). 10.1088/0031-9155/51/13/R24 16790916

[29] Attix F. H., Introduction to Radiological Physics and Radiation Dosimetry, John Wiley & Sons, Ltd (1986).

[30] Hubbell J. Seltzer S., “X-ray mass attenuation coefficients, NIST standard reference database 126,” NIST Physical Measurement Laboratory, https://www.nist.gov/pml/x-ray-mass-attenuation-coefficients (2004).

[31] Kay S. M., Fundamentals of Statistical Signal Processing: Estimation Theory, Prentice-Hall Inc. (1993).

[32] Segars W. P. et al., “4D XCAT phantom for multimodality imaging research,” Med. Phys. 37(9), 4902–4915 (2010). MPHYA6 0094-2405 10.1118/1.3480985 20964209 PMC2941518

[33] Siddon R. L., “Fast calculation of the exact radiological path for a three-dimensional CT array,” Med Phys 12(2), 252–255 (1985). MPHYA6 0094-2405 10.1118/1.595715 4000088

[34] Nouri A. Wen C., “Stainless steels in orthopedics,” in Structural Biomaterials, Woodhead Publishing Series in Biomaterials, Wen C., Ed., pp. 67–101, Woodhead Publishing (2021).

[r35] Zivic F. et al., Biomaterials in Clinical Practice: Advances in Clinical Research and Medical Devices, Springer (2017).

[36] Goharian A. Abdullah M., “Bioinert metals (stainless steel, titanium, cobalt chromium),” in Trauma Plating Systems, p. 115 (2017).

[37] Rigie D. S. La Rivière P., “An efficient material decomposition method using the Gauss-Newton algorithm,” in IEEE Med. Imaging Conf., IEEE (2015). 10.13140/RG.2.1.1680.3289

[38] Hsieh J., Computed Tomography: Principles, Design, Artifacts, and Recent Advances, Vol. 4, SPIE Press, Bellingham, Washington (2022).

[39] Wunderlich A. Noo F., “Achieving uniform noise in direct fan-beam CT reconstruction through bowtie filter design,” in IEEE Nucl. Sci. Symp. Conf. Rec., Vol. 6, pp. 4379–4382 (2007). 10.1109/NSSMIC.2007.4437083

[40] Jadick G. et al., “A scanner-specific framework for simulating CT images with tube current modulation,” Phys. Med. Biol. 66(18), 185010 (2021). 10.1088/1361-6560/ac2269 PMC8552241 34464942

[41] Rigie D. S. La Riviere P. J., “Joint reconstruction of multi-channel, spectral CT data via constrained total nuclear variation minimization,” Phys. Med. Biol. 60(5), 1741 (2015). 10.1088/0031-9155/60/5/1741 25658985 PMC4669200

[42] Rigie D. La Riviere P. Petschke A., “Spectral x-ray computed tomography reconstruction using a vectorial total variation,” US Patent 9,672,638 (2017).

[43] Star-Lack J. et al., “A piecewise-focused high DQE detector for MV imaging,” Med. Phys. 42(9), 5084–5099 (2015). MPHYA6 0094-2405 10.1118/1.4927786 26328960 PMC4529442

[r44] Rottmann J. et al., “A novel EPID design for enhanced contrast and detective quantum efficiency,” Phys. Med. Biol. 61(17), 6297 (2016). 10.1088/0031-9155/61/17/6297 27494207 PMC5525153

[r45] Hu Y.-H. et al., “Characterizing a novel scintillating glass for application to megavoltage cone-beam computed tomography,” Med. Phys. 46(3), 1323–1330 (2019). MPHYA6 0094-2405 10.1002/mp.13355 30586163

[r46] Harris T. et al., “Clinical translation of a new flat-panel detector for beam’s-eye-view imaging,” Phys. Med. Biol. 65(22), 225004 (2020). 10.1088/1361-6560/abb571 33284786 PMC9142212

[47] Harris T. et al., “Improvements in beam’s eye view fiducial tracking using a novel multilayer imager,” Phys. Med. Biol. 66(15), 155007 (2021). 10.1088/1361-6560/ac1246 PMC11102774 34233309

[48] Harris T. C. et al., “Impact of a novel multilayer imager on metal artifacts in MV-CBCT,” Phys. Med. Biol. 68(14), 145009(2023). 10.1088/1361-6560/ace09a PMC10382207 37343590

[49] Flohr T. et al., “Photon-counting CT review,” Physica Med. 79, 126–136 (2020). PHYME2 1120-1797 10.1016/j.ejmp.2020.10.030 33249223

[50] Rajendran K. et al., “First clinical photon-counting detector CT system: technical evaluation,” Radiology 303(1), 130–138 (2022). RADLAX 0033-8419 10.1148/radiol.212579 34904876 PMC8940675

[51] Zhu J. et al., “Feasibility study of three-material decomposition in dual-energy cone-beam CT imaging with deep learning,” Phys. Med. Biol. 67(14), 145012 (2022). 10.1088/1361-6560/ac7b09 35728784

[52] Lyu T. et al., “Estimating dual-energy CT imaging from single-energy CT data with material decomposition convolutional neural network,” Med. Image Anal. 70, 102001 (2021). 10.1016/j.media.2021.102001 33640721

[53] Abadi E. et al., “DukeSim: a realistic, rapid, and scanner-specific simulation framework in computed tomography,” IEEE Trans. Med. Imaging 38(6), 1457–1465 (2018). ITMID4 0278-0062 10.1109/TMI.2018.2886530 30561344 PMC6598436

[54] Sharma S. et al., “A GPU-accelerated framework for rapid estimation of scanner-specific scatter in CT for virtual imaging trials,” Phys. Med. Biol. 66(7), 075004 (2021). 10.1088/1361-6560/abeb32 PMC8381286 33652421

[55] Kaur M. et al., “Effect of scattered megavoltage x-rays on markerless tumor tracking using dual energy kilovoltage imaging,” J. Appl. Clin. Med. Phys. 24, e13993 (2023). 10.1002/acm2.13993 37071500 PMC10402669

[56] Seet K. Y. et al., “The effects of field-of-view and patient size on CT numbers from cone-beam computed tomography,” Phys. Med. Biol. 54(20), 6251 (2009). 10.1088/0031-9155/54/20/014 19794246

